# Supplementary material for: Effects of Hexagonal Boron Nitride Insulating Layers on the Driving Performance of Ionic Electroactive Polymer Actuators for Light-Weight Artificial Muscles
Source: Int J Mol Sci. 2022 Apr 29;23(9):4981. doi: 10.3390/ijms23094981 (PMC9101070; doi:10.3390/ijms23094981)
Supplement: Supplementary file 1 [file ijms-23-04981-s001.zip › ijms-1690079-supplementary/ijms-1690079-supp from proof-done.pdf]

International Journal of
Molecular Sciences

MDPI

Supplementary Materials

Effects of hexagonal boron nitride insulating layers on the driving performance of ionic electroactive polymer actuators for light-weight artificial muscles

Minjeong Park [1], Youngjae Chun [2], Seonpil Kim [3], Keun Yong Sohn [1,*], and Minhyon Jeon [1,‡]

1 Department of Nanoscience and Engineering, Center for Nano Manufacturing, Inje University, Gimhae 50834, Republic of Korea
2 Department of Industrial Engineering, Bioengineering, University of Pittsburgh, Pittsburgh, PA 15261, USA
3 Department of Military Information Science, Gyeongju University, Gyeongju 38065, Republic of Korea
* Correspondence (K. Y. S.): ksohn@inje.ac.kr; Tel.: +82-55-320-3714
‡ Correspondence (M. J.): mjeon@inje.ac.kr; Tel.: +82-55-320-3672; Fax: +82-320-3963

Characterization of ionic electroactive polymer (IEAP) actuators with hexagonal boron nitride (h-BN) insulating layers

Figures S1a and b show representative surface and cross-sectional field-emission scanning electron microscopy (FE-SEM) images, respectively, of the PEDOT:PSS/GO–Ag NW (P/GO-Ag) electrodes. An atomic force microscopy (AFM) image (Figure S1c), obtained in the non-contact mode, shows the surface roughness of the electrode. The electrical conductivity σ_e of the P/GO-Ag electrode is given by

$$\sigma_e = 1/(R_s \times t), \qquad \text{(S1)}$$

where R_s is the sheet resistance of the electrode and t is the electrode thickness; it was found to be 6250 S m^{-1}.

The low surface roughness, low and uniform sheet resistance, good electrical conductivity, and flexibility of P/GO-Ag electrodes make them suitable for use in IEAP actuators.

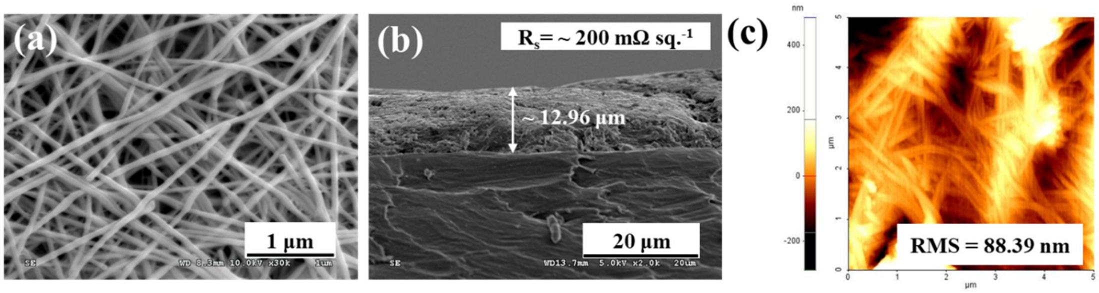

Figure S1. Morphology of PEDOT:PSS/GO-Ag NW paper electrode: (**a**) Top view FE-SEM image; (**b**) Cross-sectional FE-SEM image (R_s: sheet resistance); (**c**) AFM image and RMS surface roughness.

The surface shape and roughness of the h-BN insulating layers with different particle sizes were measured to confirm the degree of dispersion of the h-BN powder (Figure S2). The AFM images demonstrate that the h-BN powder became well-dispersed and the root mean square surface roughness of the insulating layer have less aggregation as particle size decreased.

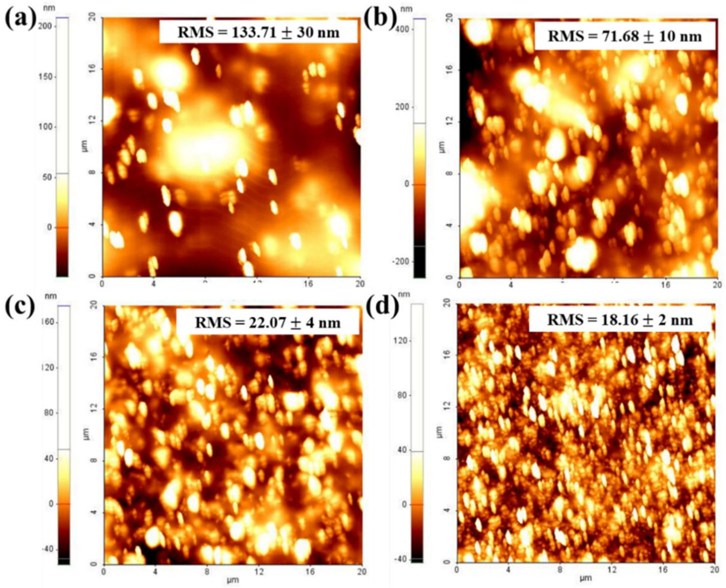

Figure S2. AFM images (20 μm × 20 μm) and RMS surface roughness of IEAP actuators with h-BN insulating layers of different particle sizes: (**a**) 1 μm; (**b**) 800 nm; (**c**) 300 nm; (**d**) 170 nm.

Electrochemical properties of IEAP actuators with h-BN insulating layers

Figures S3a and b show the average capacitances and dielectric constants of the IEAP actuators. The capacitance and dielectric constant of the IEAP actuator with h-BN−170 nm insulating layers were approximately 10^4 times higher than those of the IEAP actuator without insulating layers. In addition, the capacitance of the IEAP actuators increased with decreasing h-BN particle size. The data were collected from five repetitions using three samples to increase the reliability of the results.

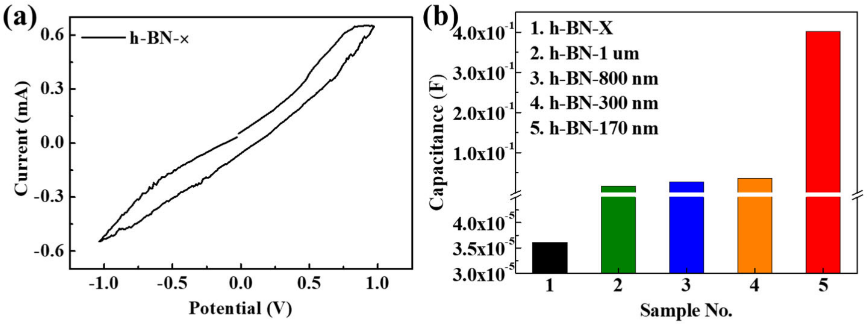

Figure S3. Average capacitances and dielectric constants of the IEAP actuators: (**a**) Cyclic-voltammetry curve of actuator without h-BN insulating layers; (**b**) Average capacitance of IEAP actuators without insulating layers (h-BN−×) and with h-BN insulating layers with different particle sizes (1 μm, 800 nm, 300 nm, and 170 nm).

The ion conductivity (σ_i) of the ionic polymer and h-BN insulating electrolytes (Figure 3 of the main text) was evaluated from

$$\sigma_i = d/(R_e \times A), \quad \text{(S2)}$$

where d is the electrolyte thickness, R_e is the electrolyte resistance of the electrode, and A is the surface area of the electrolyte.

Driving performances of IEAP actuators with h-BN insulating layers

We weighed the IEAP actuators to investigate the water uptake (WUP) of the Nafion membrane and the weight loss characteristics due to water evaporation from the Nafion membrane. WUP is defined as

$$\text{WUP} = [(W_s - W_d)/W_d] \times 100, \quad \text{(S3)}$$

where W_s and W_d are the wet and dry weights of the actuator, respectively. Table 1 shows the values of WUP and weight loss.

Figure S4 shows the driving performance of the IEAP actuator with h-BN−170 nm insulating layers. Figure S4a presents the displacement performance under five different sinusoidal input voltages (0.5, 1.0, 1.5, 2.0, and 2.5 V_{AC}) at an excitation frequency of 0.2 Hz. The displacements reached 0.40, 1.08, 1.78, 2.50, and 3.21 mm, respectively, i.e., the displacement performance improved with increasing driving voltage. The displacement performance was also measured by applying various frequencies ranging from 0.2 to 5 Hz under an input voltage of 2.5 V_{AC} (Figure S4b). Because the h-BN−170 nm powder was well-dispersed in the Nafion matrix, causing the insulating layers to form uniformly between the Nafion membrane and P/GO-Ag electrodes, the actuator movement was stable at each frequency, and its performance did not degrade. The curvature performances of IEAP actuators with h-BN−170 nm insulating layers were increase according to input voltages increase (Figure S4c). This result shows the operating efficiency of the IEAP actuators according to the applied voltages.

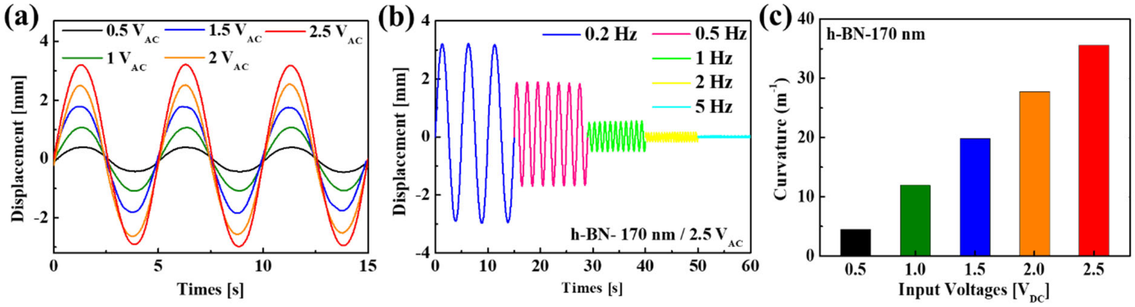

Figure S4. Driving performances of IEAP actuators with h-BN−170 nm insulating layers: (**a**) Displacement properties under various AC voltages (0.5, 1.0, 1.5, 2.0, and 2.5 V_{AC}) and fixed frequency (0.2 Hz); (**b**) Displacement properties under various frequencies (0.2, 0.5, 1, 2, and 5 Hz) and fixed voltage (2.5 V_{AC}), and (c) curvature under various input voltages (0.5, 1.0, 1.5, 2.0, and 2.5 V_{DC})

Table 2 compares the electrode sheet resistance, capacitance, and driving characteristics of various actuators from prior reports to those of the IEAP actuator with h-BN−170 nm insulating layers. Because previous researchers reported different actuator information, values of the strain (ε') are also included when available. Here,

$$\varepsilon' = 2d\delta/L^2 + \delta^2, \quad \text{(S4)}$$

where d is the thickness of the actuator cantilever, δ is the tip displacement, and L is the free length of the actuator.
